# Impact of Metabolic Syndrome on Renal and Cardiovascular Outcomes in Renal Transplant Recipients: A Single-Center Study in Japan

**DOI:** 10.3390/jcm14155303

**Published:** 2025-07-27

**Authors:** Toshihide Naganuma, Tomoaki Iwai, Daijiro Kabata, Yuichi Machida, Yoshiaki Takemoto, Junji Uchida

**Affiliations:** 1 Department of Urology, Graduate School of Medicine, Osaka Metropolitan University, Osaka 545-8586, Japan; iwai@omu.ac.jp (T.I.); ymachida@omu.ac.jp (Y.M.); p21910d@omu.ac.jp (Y.T.); uchida@omu.ac.jp (J.U.); 2 Center for Mathematical and Data Sciences, Kobe University, Kobe 657-8501, Japan; daijiro.kabata@port.kobe-u.ac.jp

**Keywords:** metabolic syndrome, renal transplantation, NCEP-ATPIII, IDF, cohort study, Japanese

## Abstract

**Background:** Several epidemiological studies have indicated that metabolic syndrome (MetS) after renal transplantation is caused by an accumulation of non-immunological risks of renal transplantation, and affects the prognosis of the kidney and the patient by increasing the risk of arteriosclerosis and cardiovascular complications. The incidence of MetS in Japanese renal transplant recipients is 14.9 to 23.8%, but its effects on cardiovascular events and kidney prognosis are not clear. Here, we report the results of a longitudinal study on MetS in renal transplant recipients. **Methods:** A retrospective cohort study was conducted in 104 stable renal transplant recipients who attended our outpatient department from January 2006 to June 2007 and were diagnosed with MetS at least 6 months after renal transplantation until 31 December 2020, or did not have MetS. The impact of MetS on composite vascular events was examined using multivariate Cox proportional hazards analysis. **Results:** The hazard ratios for the impact of MetS on composite vascular events diagnosed by NCEP Japan, NCEP Original, NCEP Asia, and IDF criteria on composite vascular events were 2.78 (95% CI: 1.15 to 6.75, *p* = 0.024), 2.65 (95% CI: 1.04 to 6.80, *p* = 0.042), 2.37 (95% CI: 0.93 to 6.01, *p* = 0.070), and 1.91 (95% CI: 0.77 to 4.75, *p* = 0.164), respectively. P for interaction was used to test the influence of each indicator, but was not statistically significant. **Conclusions:** MetS is a robust risk factor for graft loss and development of cardiovascular events in Japanese renal transplant recipients, even during long-term follow-up. This finding emphasizes the importance of monitoring and managing MetS in this population to improve long-term outcomes.

## 1. Introduction

Metabolic syndrome (MetS) is a multifactorial condition characterized by the clustering of interrelated cardiovascular and metabolic risk factors, including central obesity, hypertension, dyslipidemia, and impaired glucose tolerance [1]. These abnormalities are primarily driven by insulin resistance and chronic inflammation stemming from visceral adiposity, resulting in accelerated atherosclerosis and end-organ damage [1]. In the general U.S. population, the prevalence of MetS is reported to be 24.7–26.7% according to the original National Cholesterol Education Program Adult Treatment Panel III (NCEP-ATPIII) criteria [2,3]. By contrast, the prevalence in the Japanese general population is lower, ranging from 12.4% with the original NCEP criteria to 21.2% using the Japan-specific modifications [4]. Nevertheless, even modest metabolic disturbances have been associated with increased risks of cardiovascular disease [5], all-cause mortality [6], and the progression of chronic kidney disease [7,8].

Following renal transplantation, the risk of developing MetS may be further exacerbated by non-immunological factors such as aging, sedentary lifestyle, and diet, in addition to the metabolic adverse effects of immunosuppressive agents [9,10,11,12,13,14,15,16,17,18]. International studies have consistently shown that MetS is highly prevalent among renal transplant recipients and is associated with impaired graft function, cardiovascular morbidity, and reduced patient survival [10,15,19,20,21,22,23,24]. For example, De Vries et al. reported a 63% prevalence of MetS among Caucasian renal transplant recipients using the NCEP-ATPIII criteria [20], and Porrini et al. found a prevalence of 37.7% at four years post-transplantation in a Spanish cohort [15]. These findings highlight the heightened metabolic burden faced by transplant recipients in Western populations.

In Japan, the prevalence of MetS among kidney transplant recipients appears to be lower, estimated between 14.9% and 23.8% depending on the diagnostic criteria used [25,26]. However, the long-term impact of MetS on graft and cardiovascular outcomes in Japanese patients has not been well characterized. Ethnic differences in body composition and metabolic profiles may influence both the prevalence and clinical significance of MetS, underscoring the need for population-specific studies. Additionally, the applicability of existing diagnostic criteria, particularly those based on waist circumference, may be limited in Asian populations, where cardiometabolic risks may emerge at lower thresholds of adiposity [4,27,28].

To address these knowledge gaps, we conducted a retrospective longitudinal study in a Japanese renal transplant cohort, building upon our previous cross-sectional analysis [26]. Using four different diagnostic criteria (NCEP Japan, NCEP Original, NCEP Asia, and International Diabetes Federation [IDF]), we aimed to evaluate the prevalence of MetS and investigate its association with long-term renal and cardiovascular outcomes. This study represents one of the first efforts to assess the prognostic significance of MetS using multiple diagnostic frameworks in Japanese kidney transplant recipients over an extended follow-up period.

## 2. Subjects and Methods

### 2.1. Diagnostic Criteria for MetS

The diagnostic criteria for MetS are presented in detail in our previous publication [26] and are summarized below. This study uses NCEP-ATPIII [28] and the International Diabetes Federation (IDF) criteria [27] to diagnose MetS, based on the following diagnostic thresholds and criteria: serum triglycerides ≥ 150 mg/dL (≥1.69 mmol/L) or specific treatment for this lipid abnormality; HDL-cholesterol < 40 mg/dL (<1.04 mmol/L) for men and <50 mg/dL (<1.29 mmol/L) for women, or specific treatment for this lipid abnormality; systolic blood pressure (SBP) ≥ 130 mmHg and/or diastolic blood pressure (DBP) ≥ 85 mmHg, or use of antihypertensive medication; fasting plasma glucose ≥ 100 mg/dL (≥5.6 mmol/L) or use of antidiabetic medication; and waist circumference criteria adjusted according to geographic and ethnic considerations: Original NCEP-ATPIII criteria (NCEP Original): >102 cm for men and >88 cm for women; Modified NCEP-ATPIII criteria for Asians (NCEP Asia): ≥90 cm for men and ≥80 cm for women; Modified NCEP-ATPIII and IDF criteria for Japanese patients (NCEP Japan and IDF): ≥85 cm for men and ≥90 cm for women. In application of the criteria, for NCEP-ATPIII, MetS is diagnosed if three or more of the specified risk factors are present; while for IDF, diagnosis requires the presence of central obesity (as defined by the modified waist circumference criteria) plus any two of the other risk factors.

### 2.2. Patients

A retrospective cohort study was performed in 104 consecutive adult renal transplant recipients at Osaka Metropolitan University Hospital. Of these subjects, 101 were the same as in our previous publication [26], and 3 were added during the study inclusion period. Patients were more than 6 months post-transplant. Recipients were stratified into groups with and without MetS based upon the criteria described above. Baseline data were collected for this population between January 2006 and January 2007. The patient background, divided based on MetS diagnostic criteria, is shown in Table 1. The study protocol was conducted in accordance with the Principles of the Declaration of Helsinki and the Declaration of Istanbul, and was approved by the ethics committee of Osaka Metropolitan University Graduate School of Medicine (No. 2020-197: Approval Date (14 October 2020)). Opt-out consent was obtained instead of written informed consent, i.e., patients were provided with information explaining the proposed study (purpose, required individual data, and duration) using an information sheet or hospital website and were given the opportunity to opt out.

### 2.3. Measurements

The methods for measurement of the test values are described in detail elsewhere and are summarized below [26]. Subjects fasted overnight and arrived at the clinical research unit at 8:00 a.m., after abstaining from immunosuppressive medications. Fasting blood samples were collected to measure serum creatinine, triglycerides, HDL cholesterol, and plasma glucose. LDL cholesterol was calculated using the Friedewald formula [29]. Blood pressure was defined as the average of five automated measurements taken at 3 min intervals. Hypertension was identified through antihypertensive use, SBP ≥ 140 mmHg, or DBP ≥ 90 mmHg. Weight, height, and waist circumference were also measured. Proteinuria (+) was defined as persistent +1 or more on a urine qualitative test at the time of the outpatient visit.

### 2.4. Definition of Cardiovascular Disease at Baseline

In the present study, cardiovascular disease was defined as the presence of any of the following: brain infarction, brain hemorrhage, subarachnoid hemorrhage, angina pectoris, myocardial infarction, the need for coronary angioplasty or coronary artery bypass grafting, congestive heart failure, sudden death, valvular heart disease, severe arrhythmia, aortic aneurysm, peripheral arterial disease, and limb amputation due to vascular causes. All diagnoses were made based on established clinical and diagnostic criteria. Sudden death was defined as a witnessed death occurring within one hour of the onset of acute symptoms, in the absence of trauma or external cause.

### 2.5. Outcomes

Patients were followed retrospectively until 31 December 2020 or new onset of cardiovascular events or dialysis reintroduction, with a median follow-up period of 13.7 [8.3–14.0] years. Outcomes were cardiovascular events and death-censored renal graft loss, which was defined as reintroduction of dialysis or re-transplantation. Clinical outcome data were collected from a direct review of all medical records. All events that occurred between 1 January 2006 and 31 December 2020 (the study observation period) were collected. If there were multiple events during this period, only the first event was included. A total of 36 events were recorded: dialysis reintroduction (n = 18 cases), acute myocardial infarction (n = 1), angina pectoris (n = 3), percutaneous coronary intervention (n = 4), valvular surgery (n = 1), severe arrhythmia (n = 1), aortic aneurysm (n = 1), peripheral arterial disease (n = 3), limb amputation (n = 1), brain hemorrhage (n = 1), cerebral infarction (n = 1), and sudden death (n = 1).

### 2.6. Statistical Analysis

Baseline characteristics were summarized using medians and interquartile ranges for continuous variables and percentages and counts for categorical variables. All statistical analyses were conducted with a two-sided significance level of 5%, except for interaction terms. Because of the underpowered nature of the interaction analysis, we used a 20% significance level for hypothesis testing of these terms. All analyses were performed using R ver. 4.4.2 (https://www.r-project.org/foundation/ (accessed on 14 July 2025)) with the “rms” and “RcmdrPlugin.EZR” packages. Kaplan–Meier curves for survival analysis were tested for significant differences by log-rank test. Multivariate Cox proportional hazards regression analysis was performed to examine the association between MetS and composite vascular events in renal transplant recipients, with adjustment for all covariates listed in Table 1. Four indices were used to assess MetS, and a power term between the variable indicating the presence or absence of MetS and the variable indicating the indices used to measure MetS (four types) was taken into account in the model to evaluate the relationship between MetS and the event as determined by each index, and to examine differences in the relationship between the indices. Because repeated measures data were used, with one row for each indicator in each case, intra-case correlations were adjusted for using the Huber–White variance–covariance matrix.

## 3. Results

### 3.1. Diagnosis of MetS Using Four Criteria

At baseline, the rates of MetS using the NCEP-ATPIII (Japan, Original, Asia) and IDF criteria were 24.0%, 14.4%, 22.1%, and 16.3%, respectively (Table 1).

### 3.2. Cumulative Event-Free Survival Probability Using Each Set of Diagnostic Criteria for Mets

**Figure 1:**
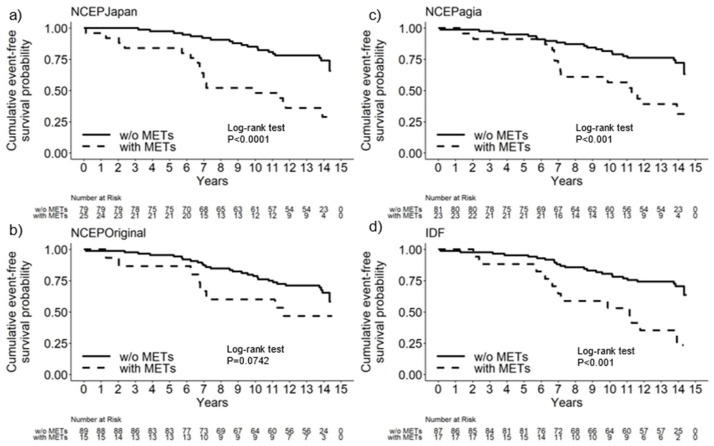
Kaplan–Meier event-free survival curves for cases with and without MetS based on four sets of diagnostic criteria for MetS. (**a**) NCEP Japan, (**b**) NCEP Original, (**c**) NCEP Asia, (**d**) IDF.

Kaplan–Meier curves comparing event-free survival in the presence or absence of MetS are shown in Figure 1, based on the NCEP Japan (*p* < 0.0001), NCEP Original (*p* = 0.0742), NCEP Asia (*p* < 0.001), and IDF (*p* < 0.001) diagnostic criteria for MetS.

### 3.3. Impact of MetS on Composite Vascular Events Depends on Diagnostic Criteria

The hazard ratios for the impact of MetS diagnosed by NCEP Japan, NCEP Original, NCEP Asia, and IDF criteria on composite vascular events (Table 2) were 2.78 (95% CI: 1.15 to 6.75, *p* = 0.024), 2.65 (95% CI: 1.04 to 6.80, *p* = 0.042), 2.37 (95% CI: 0.93 to 6.01, *p* = 0.070), and 1.91 (95% CI: 0.77 to 4.75, *p* = 0.164), respectively. P for interaction was used to test the influence of each indicator, but none reached statistical significance (*p* = 0.37).

## 4. Discussion

This longitudinal study expands upon our previous cross-sectional study of metabolic syndrome (MetS) in renal transplant recipients. It provides new, long-term evidence that MetS diagnosed in the chronic phase after transplantation is an independent predictor of adverse clinical outcomes in a Japanese cohort [26]. Over a median follow-up of nearly 14 years, MetS conferred a two- to threefold increased risk of composite vascular events including cardiovascular disease and graft loss compared to those without MetS. To our knowledge, this is the first Japanese study to demonstrate the long-term prognostic implications of MetS in renal transplant recipients.

MetS is a cluster of metabolic abnormalities (central obesity, hypertension, dyslipidemia, and impaired glucose metabolism) that promote atherosclerosis and chronic kidney disease [1]. These components are interlinked through underlying mechanisms such as insulin resistance and visceral fat accumulation, which drive inflammation and endothelial dysfunction [1]. In the general Japanese population, the prevalence of MetS is lower than in Western populations [4], yet its impact on organ damage and mortality appears to be comparable or even greater [2,5,7]. Our findings support the idea that in Japanese renal transplant recipients, even modest metabolic disturbances can have significant long-term consequences.

The post-transplant environment introduces additional metabolic risks. Immunosuppressive agents such as corticosteroids, calcineurin inhibitors, and mTOR inhibitors are known to contribute to post-transplant hypertension, dyslipidemia, glucose intolerance, and central adiposity [9,10,11,12,13,14,15,16,17,18]. Consequently, the development of MetS in transplant recipients represents both a continuation of preexisting cardiometabolic trends and an iatrogenic complication. Notably, in our cohort, many patients fulfilled MetS criteria despite having waist circumferences below the traditional thresholds. This discrepancy suggests that reliance on standard cutoffs for central obesity may underestimate risk in Asian populations [26]. Among the four diagnostic definitions employed in our study, the NCEP Japan criteria identified the highest number of MetS cases and demonstrated the strongest association with adverse outcomes, supporting the appropriateness of ethnic-specific diagnostic adaptations [4,28].

Our results are consistent with those of a recent Korean nationwide cohort study, which reported a higher MetS prevalence (~40%) after transplantation and showed that pre-existing and de novo MetS were associated with increased risks of cardiovascular events, graft loss, and mortality [22]. That study used body mass index (BMI) instead of waist circumference in its diagnostic algorithm, reflecting regional practices and possibly offering greater applicability in East Asian populations. Despite methodological differences and shorter follow-up periods, the strength of the associations observed in both studies reinforces the significance of MetS as a key modifiable risk factor after kidney transplantation.

Several clinical implications arise from our findings. First, the identification of MetS provides a target for early intervention in transplant care. Pharmacologic control of blood pressure, lipids, and glucose; adjustment of immunosuppressive protocols; and lifestyle counseling may mitigate the risk of graft failure and cardiovascular morbidity [9,11,12,13,14,16,17]. Second, our data support routine screening for MetS in transplant recipients, using criteria that are sensitive to ethnic and population-specific risk profiles. Third, our findings call for the development of interventional studies aimed at reversing MetS after transplantation and assessing the effect of these interventions on long-term outcomes [13,16].

Several limitations must be acknowledged. The relatively small sample size, despite long-term follow-up, limited our ability to analyze cardiovascular and renal outcomes separately. Additionally, the retrospective nature of the study introduces the possibility of confounding by changes in clinical practice over time, including the use of newer antidiabetic or lipid-lowering agents and variations in immunosuppressive regimens. In fact, mTOR inhibitors were not used at baseline. Patients diagnosed with MetS may have received more intensive monitoring or interventions, which could attenuate the observed associations. Despite these limitations, the consistency and magnitude of the hazard ratios across all definitions of MetS strengthen the validity of our findings.

In conclusion, MetS is a robust risk factor for graft loss and development of cardiovascular events in Japanese renal transplant recipients, even during long-term follow-up. Given its modifiable nature and rising prevalence, systematic screening and tailored management of MetS should be incorporated into long-term transplant care strategies. Further prospective, multicenter studies are needed to validate diagnostic thresholds and evaluate the efficacy of targeted interventions in improving outcomes for this vulnerable population.

## Figures and Tables

**Table 1:** Diagnosis of MetS using four criteria and patient background.

		Modified NCEP-ATPIII Criteria for Japanese		Original NCEP-ATPIII Criteria		Modified NCEP-ATPIII Criteria for Asians		IDF Criteria for Japanese	
	Level	No	Yes	No	Yes	No	Yes	No	Yes
N		79	25	89	15	81	23	87	17
Underlying diseases % (freq)	Diabetic Nephropathy	2.5 (2)	16.0 (4)	5.6 (5)	6.7 (1)	2.5 (2)	17.4 (4)	3.4 (3)	17.6 (3)
	Others	97.5 (77)	84.0 (21)	94.4 (84)	93.3 (14)	97.5 (79)	82.6 (19)	96.6 (84)	82.4 (14)
Age (median [IQR])	years	41.00 [35.00, 53.00]	54.00 [47.00, 56.00]	45.00 [35.00, 54.00]	52.00 [43.00, 55.50]	43.00 [35.00, 53.00]	55.00 [47.00, 56.00]	43.00 [35.00, 54.00]	54.00 [51.00, 56.00]
Sex % (freq)	female	43.0 (34)	12.0 (3)	38.2 (34)	20.0 (3)	40.7 (33)	17.4 (4)	41.4 (36)	5.9 (1)
	male	57.0 (45)	88.0 (22)	61.8 (55)	80.0 (12)	59.3 (48)	82.6 (19)	58.6 (51)	94.1 (16)
eGFR (median [IQR])	mL/min/1.73 m^2^	49.23 [40.88, 57.37]	46.41 [38.39, 51.05]	49.23 [40.37, 57.10]	43.88 [39.65, 54.33]	48.55 [40.01, 56.57]	49.60 [41.22, 55.84]	48.55 [39.75, 57.37]	49.60 [42.29, 53.21]
CVD history % (freq)	absence	91.1 (72)	76.0 (19)	87.6 (78)	86.7 (13)	88.9 (72)	82.6 (19)	90.8 (79)	70.6 (12)
	presence	8.9 (7)	24.0 (6)	12.4 (11)	13.3 (2)	11.1 (9)	17.4 (4)	9.2 (8)	29.4 (5)
CRP (median [IQR])	mg/dL	0.02 [0.01, 0.04]	0.05 [0.04, 0.11]	0.02 [0.01, 0.06]	0.04 [0.03, 0.06]	0.02 [0.01, 0.05]	0.04 [0.04, 0.10]	0.02 [0.01, 0.05]	0.05 [0.03, 0.14]
Proteinuria % (freq)	absence	88.6 (70)	76.0 (19)	86.5 (77)	80.0 (12)	87.7 (71)	78.3 (18)	87.4 (76)	76.5 (13)
	presence	11.4 (9)	24.0 (6)	13.5 (12)	20.0 (3)	12.3 (10)	21.7 (5)	12.6 (11)	23.5 (4)
Hemodialysis duration (median [IQR])	years	2.00 [0.83, 6.50]	3.00 [1.43, 4.35]	2.08 [1.00, 5.80]	3.67 [1.80, 9.34]	2.03 [0.92, 6.25]	3.00 [1.38, 6.07]	2.26 [0.88, 6.33]	3.11 [1.43, 4.35]
CNI % (freq)	cyclosporine	57.0 (45)	80.0 (20)	59.6 (53)	80.0 (12)	55.6 (45)	87.0 (20)	58.6 (51)	82.4 (14)
	tacrolimus	43.0 (34)	20.0 (5)	40.4 (36)	20.0 (3)	44.4 (36)	13.0 (3)	41.4 (36)	17.6 (3)
AR % (freq)	absence	77.2 (61)	84.0 (21)	78.7 (70)	80.0 (12)	76.5 (62)	87.0 (20)	78.2 (68)	82.4 (14)
	presence	22.8 (18)	16.0 (4)	21.3 (19)	20.0 (3)	23.5 (19)	13.0 (3)	21.8 (19)	17.6 (3)
Donor % (freq)	living donor	83.5 (66)	56.0 (14)	83.1 (74)	40.0 (6)	84.0 (68)	52.2 (12)	81.6 (71)	52.9 (9)
	deceased donor	16.5 (13)	44.0 (11)	16.9 (15)	60.0 (9)	16.0 (13)	47.8 (11)	18.4 (16)	47.1 (8)
LDL (median [IQR])	mg/dL	113.20 [91.50, 131.60]	114.20 [95.00, 131.20]	113.60 [92.80, 131.80]	112.60 [92.80, 120.00]	111.20 [91.20, 131.40]	116.20 [99.70, 131.60]	113.60 [91.70, 130.50]	112.60 [95.00, 132.00]
Post-transplant duration (median [IQR])	years	3.98 [2.33, 9.73]	6.31 [2.21, 9.97]	4.14 [2.29, 9.59]	5.11 [2.24, 12.46]	4.14 [2.38, 9.3]	5.11 [2.14, 10.92]	4.14 [2.33, 9.9]	5.11 [2.06, 9.63]
Uric acid level (median [IQR])	mg/dL	6.7 [5.6, 7.9]	7.1 [6.7, 8.0]	6.8 [5.6, 7.9]	7.1 [6.8, 8.1]	6.7 [5.5, 7.8]	7.2 [6.8, 8.1]	7.0 [5.6, 8.0]	7.1 [6.7, 7.9]
Calcium level (median [IQR])	mg/dL	9.7 [9.50, 10.00]	9.8 [9.50, 10.30]	9.7 [9.50, 10.00]	9.9 [9.75, 10.35]	9.7 [9.50, 10.00]	9.9 [9.35, 10.30]	9.7 [9.50, 10.00]	9.9 [9.50, 10.30]
Phosphorus level (median [IQR])	mg/dL	3.1 [2.7, 3.4]	3.1 [2.7, 3.6]	3.1 [2.7, 3.4]	3.1 [2.6, 3.5]	3.1 [2.7, 3.4]	3.0 [2.7, 3.5]	3.1 [2.7, 3.4]	3.2 [2.7, 3.5]
Use of antiplatelet drugs % (freq)	No	77.2 (61)	68.0 (17)	76.4 (68)	66.7 (10)	77.8 (63)	82.6 (19)	74.7 (65)	76.5 (13)
	Yes	22.8 (18)	32.0 (8)	23.6 (21)	33.3 (5)	22.2 (18)	17.4 (4)	25.3 (22)	23.5 (4)

NCEP-ATPIII, National Cholesterol Education Program Adult Treatment Panel III; IDF, International Diabetes Federation; IQR, interquartile range; eGFR, estimated Glomerular Filtration Rate; CVD, cardiovascular disease; CRP, C-reactive Protein; CNI, calcineurin inhibitor; AR, Acute Rejection; LDL, low-density lipoprotein.

**Table 2:** Effect of MetS on composite vascular events based on diagnosis of MetS using four sets of criteria.

Scale	HR	95% Confidence Interval		*p*	*p* for Interaction
		Lower	Upper		
NCEP-ATPIIIJapan	2.78	1.15	6.75	0.024	0.371
NCEP-ATPIIIOriginal	2.65	1.04	6.8	0.042	
NCEP-ATPIIIagia	2.37	0.93	6.01	0.07	
IDF	1.91	0.77	4.75	0.164	

MetS, metabolic syndrome; HR, hazard ratio: NCEP-ATPIII, National Cholesterol Education Program Adult Treatment Panel III; IDF, International Diabetes Federation. All covariates listed in the background table have been adjusted for.

## Data Availability

The data presented in this study are available on request from the corresponding author.
